# Suicide Risk and Association With the Different Trauma During the COVID-19 Pandemic Period: A Cross-Sectional Study on Adolescent With Different Learning Stage in Chongqing, China

**DOI:** 10.3389/fpubh.2022.858157

**Published:** 2022-04-28

**Authors:** Yao Yu, Tingting Wu, Shanshan Wang, Weiwei Liu, Xin Zhao

**Affiliations:** ^1^Faculty of Education, Southwest University, Chongqing, China; ^2^Collaborative Innovation Platform of 0-6-Year-Old Children's Development and Education Network, Chongqing University of Education, Chongqing, China; ^3^Collaborative Innovation Center for Student Growth and Development, Chongqing University of Education, Chongqing, China; ^4^Chongqing Collaborative Innovation Center for Functional Food, Chongqing University of Education, Chongqing, China; ^5^Department of Food and Nutrition, College of Medical and Life Sciences, Silla University, Busan, South Korea; ^6^School of Public Health and Management, Chongqing Medical University, Chongqing, China; ^7^Research Center for Public Health Security Chongqing Medical University, Chongqing, China

**Keywords:** adolescent, suicide risk, trauma, family, COVID-19 pandemic

## Abstract

This study aimed to examine the current suicidal risk and whether the suicidal risk was associated with a wide range of trauma. The self-administered online questionnaire was adopted to collect suicide risk (SR) such as suicidal ideation, self-harm, suicide attempts, and different trauma information of the adolescents by cluster sampling in Chongqing, China. Multivariable linear regression was presented to assess the association between different risks of trauma and SR scores. Approximately 14.7% of adolescents enrolled reported suicide ideation and more than 10% of adolescents have experienced one kind of trauma during the Coronavirus disease 2019 (COVID-19) pandemic period. After adjusting for confounding variables, adolescents who suffered family hurt had a higher risk score of SR (beta coefficients (β) = 0.289, 95% confidence interval (*CI*) = 0.115–0.463). A positive association was found among participants from junior and senior school (β = 0.415, 95% *CI* = 0.152, 0.768), and the SR score was positively associated with sexism among participants from the university/college (β = 0.238, 95% *CI* = 0.042, 0.434). The most potentially obvious trauma that contributed to SR in junior and senior school adolescents might be cyberbullying. Family neglect or abuse might be a detrimental factor in SR for adolescents whether those in junior school or those in university school in China. More interventions, like education related to cyberbullying and family abuse, should be prioritized to reduce the risk of suicide.

## Introduction

The Coronavirus disease 2019 (COVID-19) has profoundly caused the global public health crisis around the world since it broke out. The threat is continuing to ferment with the virus of COVID-19 constantly mutating, the virus Omicron strain has been recurring all over the world (1). According to the estimation of the United Nations Educational, Scientific and Cultural Organization, many countries have experienced national school closures related to COVID-19 (2). This situation made the children and adolescents have been exceptionally affected by these situations. The social contacts were strongly limited and out-of-home leisure time activities were canceled. Parents were asked to support their children with homeschooling, while at the same time working from home. External support by other family members and social support systems has fallen away. This has put a lot of pressure on adolescents and their families, which could result in distress, mental health problems, and violence (3). The mental health effects of the COVID-19 pandemic might beprofound, and the suicide rates might rise (4).

Suicide is a serious global public health issue, especially in adolescents. The report of the World Health Organization (WHO) shows that close to 800,000 people die by suicide every year, which become among the top twenty leading causes of death worldwide and was the second leading cause of death between 15 and 29 years of age (5). In China, suicide has become the leading cause of death among Chinese young adults, accounting for 19% of all deaths (6). Although the overall suicide rate in China decreases significantly over the past decade, rates in young people 15–24 years of age do not reduce (7, 8). The prevalence of suicidal ideation (SI, thoughts and plans of ending one's life) and suicide attempts (SA, engagement in potentially self-injurious behavior that does not result in death) among Chinese adolescents was 23 and 4%, respectively (9). In response to the high prevalence of suicide, risk factors for SI and suicidal behavior have been identified as diverse, which range from psychopathology to interpersonal adversity, such as trauma (10).

Trauma is a complex emotional response to a stressful event that overwhelms the individual's capacity to cope. All traumas or stresses that can upset the psychophysical well-being of an individual, such as economic problems, diseases associated with chronic pain, war, sexual violence, grief, bullying, family conflicts, and other violence, are risk factors (11, 12). The COVID-19, created severe trauma in the population from a health, economic, political, and social point of view, causing a radical change in everyone's daily life and relation to the external environment, such as the economic situation has worsened with high and rising levels of unemployment in all affected countries (9, 13). Thus, exploring the association between trauma and suicide is likely to become a more pressing consideration as the pandemic spreads and has longer-term effects on adolescents to prevent suicide during the epidemic recovery period.

The suicide risk (SR) assessment is the process of assessing and analyzing individuals who may have suicidal tendencies and risks, and then screening out the high-risk individuals, which can prevent suicide to a certain extent (14). Therefore, the accurate assessment of SR is a key link in suicide prevention, which can provide a basis for subsequent intervention and create opportunities to prevent suicide.

According to the data of the *China Statistical Yearbook*, China's adolescents (15–24 years old) population accounts for 10.48% of the total population (15). To provide reserves for the rapid development of the country, it is particularly important to pay attention to the healthy development of adolescents. Chongqing, China is located in the southwestern region of China, where the economy has been developing rapidly in recent years. Due to its special geographical location, adolescents in Chongqing are relatively less stressed than adolescents from coastal cities. However, research data show that 38.8–54.0% of Chongqing adolescents have mental health problems (16, 17). Thus, this study focused on the SR among different grade adolescents in Chongqing, China during the COVID-19 pandemic period, and the correlation between trauma and the SR was discussed. This could be beneficial to prevent youth suicide and contribute to reducing the SR in adolescents.

## Methods and Materials

### Methods and Participants

This is a cross-sectional design. The participants were middle school (senior and junior school, grade 7–12th) and university/college students. A stratified sampling frame was used according to the development of socio-economic, regional differences, urban and rural types, and other factors. Two districts and two counties were selected from Chongqing city through a convenience sampling method. In each district or county, one middle school or one university was selected using a random number table. In each school, one class was selected from each grade using a random number table. All students in selected classes were recruited through a questionnaire survey.

Due to the COVID-19 epidemic, this survey was conducted by online questionnaire during September 2020. The questionnaires were completed online through WeChat and website link, and a total of 1,800 valid questionnaires were collected, due to the number of primary school students being small, they were excluded. Finally, the number of valid questionnaires included in this study is 1,248. Also, this study was approved by the Ethics Committee Review Committee of Chongqing Collaborative Innovation Center for Functional Food in Chongqing University of Education (202009HS01). The written informed consent to participate in this study was provided by the participant's legal guardian.

### Measurement of Suicidal Risk

The Chinese Youth Health-Related Behavior Questionnaire that was corrupted by the Chinese Center for Disease Control and Prevention (CDC) (18). was used. The information about suicidal thoughts and attempts, physical and mental health, health-related behaviors, support of family and school was captured.

Beyond suicidal thoughts and suicide plans, there are several behaviors in which there is an intention to die, such as depression emotion, SA, interrupted attempts, aborted attempts, and other suicidal preparatory acts. Suicidal behaviors require, not only the self-harm act but also there must be suicidal intent. By contrast, when individuals engage in self-injurious behaviors for reasons other than ending their lives, this behavior is termed as non-suicidal self-injury. Deliberate self-harm behaviors comprise self-injurious behaviors regardless of intentionality. Thus, the suicidal risk (SR) was measured by the following seven questions in this study: ‘During the past 2 weeks or more time, did you feel sad and desperate to be unable to carry out daily activities?' (yes/no); ‘did you approve of the behavior of suicide?' (yes/no); During the past 12 months, did you hurt yourself?'(yes/no); ‘During the past 12 months, did you seriously consider suicide?'(yes/no); ‘During the past 12 months, did you make a suicide plan?' (yes/no); and ‘During the past 12 months, did you attempt suicide?' (yes/no), if the answer to this question is yes, the participants would be asked to answer ‘how many times did you attempt suicide or did you make a suicide plan?' (none/once/2–3 times/4–5 times/≥6 times). As for the first five questions, if the answer is yes, the suicidal risk score gets 1 point. otherwise, 0 points were counted. The answer to the sixth question was got 0 to 4 points. When the sixth question answer is yes, none = 0, once = 1, 2–3 times =2, 4–5 times = 3, ≥5 times = 4; and the sixth question answer is no, the score is also 0. The score of suicidal risk is higher, the suicidal risk was higher.

### Variables of Sociodemographic

To adjust for potential confounders, the gender, nationality, grade, regions, living conditions, and family support were set as socio-demographic characteristics variables. The nationality was categorized as ‘Han' and ‘minority'; the grade was categorized as the junior school, the senior school, and the university/college; the regions were classified into two levels, urban and rural. The living conditions of participants included whether they were stay-at-home or migrant adolescents. The family support was scored by assessing the relationships of participants with their parents.

### Lifestyle and Health Status

The lifestyle consists of a history of smoking, drinking, and exercise status. The health status variables include a history of the diseases, which were dichotomized and defined by doctors, such as loneliness, sleep quality, and physical status. Loneliness was a subjective feeling; wand classified it into three levels, such as low, medium, and high level. The sleep quality was categorized into three levels, such as low, medium, and high level by assessing the participants' problems when they were sleeping. The physical status was measured by the following questions ‘Did you have the following physical problems in the last 2 weeks? headache/stomachache/back pain/depressed/irascibility/nervous/difficulty of fall asleep/dizzy)'?

### Variables of Trauma Risk

The trauma risk (TR) in this study was categorized into six facets, family abuse or neglect, bullying at school, sexual assault, sexism, economic discrimination, and cyberbullying. The ignorance of parents, the cures and injury by parents, the violence between parents were concluded in the family abuse or neglect. The bullying at school was measured by asking the participants whether they used to be given a nickname, or be teased, or be sidelined, or be disrespectful, or be bullied by teachers or their schoolmates/classmates. Sexual assault was an incident that involves sexual contact or language violation that is forced by somebody, and the sexism and economic discrimination were scored by asking percipients whether they used to get sexism or economic discrimination by anybody. Participants who received any trauma in one specific aspect, are considered to have trauma of this specific aspect. Otherwise, it is regarded as no trauma in this specific respect.

### Statistical Analyses

All statistical data were analyzed using two-sided *t*-tests in Stata statistical software (Stata, version 15.1, Cary, NC, USA). Considering the adolescents in university/college and junior and senior school youths may be at different physiological stages and may face different social pain, we analyzed the grade as the subgroup. The ANOVA test was used for the continuous variables and the chi-square analysis was used to estimate the different trauma among adolescents experiencing SI and those with a history of SAs. Multivariable linear regression was presented to assess the association between different risks of trauma and SR scores. To adjust for potential confounders, the following three models were used. Model 1 adjusted for variables of the different trauma, bullying at school, sexual assaults violence, sexism, economic discrimination, and cyberbullying. Model 2 further adjusted for socio-demographic characteristics variables which included gender, nationality, regions, living conditions, and family support. Model 3 further adjusted for variables of lifestyle and health status. The positive regression coefficient indicates an increase in suicidal risk scores. Results are reported for beta coefficients (β), and 95% confidence interval (95% *CI*).

## Results

The characteristics of the 1,248 participants in the study population were presented in Table 1. The mean age of the participants was 16.80 years old and more than two-thirds (76.28%) showed that have a close relationship with their parents. More than 1 in 10 participants (14.34%) reported the SI in the last 12 months and 8.25% had done a plan to suicide. Besides, 8.17% of participants attempted suicide in their lifetime. Approximately, more than 10% (12.1%) of participants did self-mutilation behavior. As for the lifestyle, more than almost three-quarters of participants (75.24%) were lack of exercise during the survey phase. Nearly one-third of adolescents (28.53%) have drunk once or more times. There were 8.03% of participants with a history of smoking and the prevalence of smoking and drinking among college/university students was higher than those of junior and senior school youth, significantly (*p* < 0.05). As for the health status, the participants in the junior and senior schools have higher aloneness than the adolescents in university/college (32.01 vs. 26.06%, *p* < 0.05). Besides, the participants in junior and senior school may have lower physical health (5.83 vs. 5.14%, *p* < 0.05).

**Table 1:** Suicidal risk and characteristics of adolescents.

**Variables**		**Group 1 (*N* = 703)**	**Group 2 (*N* = 545)**	**Total (*N* = 1,248)**	***P*-value**
**Suicidal risk variables**
Suicidal ideation	Yes	138 (19.63%)	41 (7.52%)	179 (14.34%)	<0.001
	No	565 (80.37%)	504 (92.48%)	1,069 (85.66%)	
suicidal plan	Yes	84(11.95%)	19(3.49%)	103 (8.25%)	<0.001
	No	619(88.05%)	526(96.51%)	1,145 (91.75%)	
Suicidal attempt	Yes	79 (11.24%)	23 (4.22%)	102 (8.17%)	<0.001
	No	624 (88.76%)	522 (95.78%)	1,146 (91.83%)	
Self-mutilation	Yes	115 (16.36%)	36 (6.61%)	151 (12.10%)	<0.001
	No	588 (83.64%)	509 (93.39%)	1,097 (87.90%)	
**Sociodemographic variables**					
Age		15.00 ± 1.55	19.13 ± 1.09	16.80 ± 2.46	<0.001
Gender	Male	301 (42.82%)	116 (21.28%)	417 (33.41%)	<0.001
	Female	402 (57.18%)	429 (78.72%)	831 (66.59%)	
Nationality	Han	690 (98.15%)	487 (89.36%)	1,177 (94.31%)	<0.001
	Minority	13 (1.85%)	58 (10.64%)	71 (5.69%)	
Regions	Urban	384 (54.62%)	183 (33.58%)	567 (45.43%)	<0.001
	Rural	319 (45.38%)	362 (66.42%)	681 (54.57%)	
Leftover children	Yes	86 (12.23%)	/	86 (6.89%)	/
	No	617 (87.77%)	/	617 (49.44%)	
Immigrant children	Yes	93 (13.23%)	/	93 (7.45%)	/
	No	610 (86.77%)	/	610 (48.88%)	
Relationship with parents	Closed	523 (74.40%)	429 (78.72%)	952 (76.28%)	0.180
	Moderate	153 (21.76%)	101 (18.53%)	254 (20.35%)	
	Unclosed	27 (3.84%)	15 (2.75%)	42 (3.37%)	
**Lifestyle and health status**					
Lack of exercise	Yes	487 (69.27%)	452 (82.94%)	939 (75.24%)	<0.001
	No	216 (30.73%)	93 (17.06%)	309 (24.76%)	
History of smoking	Yes	42 (5.97%)	59 (10.83%)	101 (8.09%)	0.002
	No	661 (94.03%)	486 (89.17%)	1,147 (91.91%)	
History of drinking	Yes	141 (20.06%)	215 (39.45%)	356 (28.53%)	<0.001
	No	562 (79.94%)	330 (60.55%)	892 (71.47%)	
History of disease	Yes	24 (3.41%)	18 (3.30%)	42 (3.37%)	0.910
	No	679 (96.59%)	527 (96.70%)	1,206 (96.63%)	
Loneliness	Low level	270 (38.41%)	200 (36.70%)	470 (37.66%)	0.009
	Medium level	208 (29.59%)	203 (37.25%)	411 (32.93%)	
	High level	225 (32.01%)	142 (26.06%)	367 (29.41%)	
Sleep quality	High level	222 (31.58%)	161 (29.54%)	383 (30.69%)	0.660
	Medium level	317 (45.09%)	259 (47.52%)	576 (46.15%)	
	Low level	164 (23.33%)	125 (22.94%)	289 (23.16%)	
Physical health	High level	472 (67.14%)	401 (73.58%)	873 (69.95%)	0.045
	Medium level	190 (27.03%)	116 (21.28%)	306 (24.52%)	
	Low level	41 (5.83%)	28 (5.14%)	69 (5.53%)	

*Data are presented as Mean ± SD for continuous measures, and n (%) for categorical measures. Group 1: the adolescents in junior and senior school; Group 2: the adolescents in university/college*.

### Risk of Trauma Among Adolescents With Different Suicidal Risk Behavior

Table 2 displayed the risk of trauma among adolescents experiencing suicide ideation and those with a history of SA during the COVID-19 pandemic period. Nearly 1 in 3 adolescents (26.52%) suffered family neglect or abuse, and the prevalence of participants who have suffered bullying at school, sexual assault, sexism, economic discrimination, and other social harm is 36.38, 12.18, 33.25, 26.60, and 16.9%, respectively. The adolescents who experienced SI were suffered more social hurt risk than those were not experienced suicide ideation and there were significant differences among them (*p* < 0.05) (Table 2).

**Table 2:** The different trauma among adolescents experiencing suicidal ideation and those with a history of the suicide attempt.

**Social hurt risk variables**			**Suicidal ideation**			**Suicidal attempt**		
		**Total**	**Yes**	**No**	***P*-value**	**Yes**	**No**	***P-*value**
Family neglect or abuse	Yes	331 (26.52%)	99 (55.31%)	232 (21.70%)	<0.001	66 (64.71%)	265 (23.12%)	<0.001
	No	917 (73.48%)	80 (44.69%)	837 (78.30%)		36 (35.29%)	881 (76.88%)	
Bullying at school	Yes	454 (36.38%)	114 (63.69%)	340 (31.81%)	<0.001	69 (67.65%)	385 (33.60%)	<0.001
	No	794 (63.62%)	65 (36.31%)	729 (68.19%)		33 (32.35%)	761 (66.40%)	
Sexual assault	Yes	152 (12.18%)	45 (25.14%)	107 (10.01%)	<0.001	27 (26.47%)	125 (10.91%)	<0.001
	No	1,096(87.82%)	134(74.86%)	962 (89.99%)		75 (73.53%)	1,021(89.09%)	
Sexism	Yes	415 (33.25%)	95 (53.07%)	320 (29.93%)	<0.001	60 (58.82%)	355 (30.98%)	<0.001
	No	833 (66.75%)	84 (46.93%)	749 (70.07%)		42 (41.18%)	791 (69.02%)	
Economic discrimination	Yes	332 (26.60%)	68 (37.99%)	264 (24.70%)	<0.001	49 (48.04%)	283 (24.69%)	<0.001
	No	916 (73.40%)	111(62.01%)	805 (75.30%)		53 (51.96%)	863 (75.31%)	
Cyberbullying	Yes	211 (16.91%)	76 (42.46%)	135 (12.63%)	<0.001	49 (48.04%)	162 (14.14%)	<0.001
	No	1,037 (83.09%)	103(57.54%)	934 (87.37%)		53 (51.96%)	984 (85.86%)	

*Data are presented as n (%) for categorical measures*.

### Associations Between Suicidal Risk and Trauma

Multivariable adjusted the associations between SR scores and different traumas were shown in Table 3. In model 1, all participants suffered family neglect or abuse had a higher score of SR (β = 0.631, 95% *CI* = 0.454–8.08), and the associations remained significant after adjusting for confounding variables in model 2 and the model3 (β = 0.420, 95% *CI* = 0.240–0.601; and β = 0.289, 95% *CI* = 0.115–0.463). The same results were found among participants in junior and senior school, with beta coefficients ranging from 0.29 (95% *CI* = 0.320, 0.552) for family neglect or abuse to 0.415 (95% *CI* = 0.152, 0.768) for cyberbullying. The SR score was positively associated with cyberbullying in all three models (*p* < 0.05). In fully adjusted models (model 3), there was no evidence that the SR was associated with bullying at school (*p* = 0.19), sexual assault (*p* = 0.399), sexism (*p* = 0.161), and economic discrimination (*p* = 0.755). However, we found that the SR score of the participants from the university/college was positive with sexism. Additionally, the SR score was not significantly associated with cyberbullying among participants from the university/college (*p* > 0.05) while a positive association was found among participants from the junior and senior schools.

**Table 3:** Association (beta coefficient) between SHS and suicide risk score by multiple linear regression among different grade adolescents.

**Variables**	**Model**			**Model 2**			**Model 3**		
	**Group 1**	**Group 2**	**All**	**Group 1**	**Group 2**	**All**	**Group 1**	**Group 2**	**All**
**Family neglect or abuse**									
Yes	0.744^**^ **(0.478, 1.01)**	0.364^**^ **(0.163, 0.566)**	0.631^**^ **(0.454, 0.808)**	0.516^**^ **(0.243, 0.79)**	0.254^*^ **(0.05, 0.458)**	0.42^**^ **(0.24, 0.601)**	0.292^*^ **(0.032, 0.552)**	0.243^*^ **(0.043, 0.443)**	0.289^**^ **(0.115, 0.463)**
**Bullying at school**									
Yes	0.194 (−0.068, 0.456)	0.026 (−0.160, 0.212)	0.173^**^ (0.003, 0.343)	0.253 (−0.004, 0.51)	0.015 (−0.166, 0.196)	0.167^**^ (0.001, 0.332)	–	−0.02 (−0.199, 0.158)	0.107 (−0.053, 0.266)
**Sexual assault**									
Yes	0.427^**^ (0.040, 0.814)	0.123 (−0.107, 0.353)	0.21^*^ (−0.021, 0.441)	0.436^**^ (0.058, 0.814)	0.122 (−0.103, 0.346)	0.264^**^ (0.038, 0.491)	0.217 (−0.137, 0.571)	0.022 (−0.203, 0.247)	0.094 (−0.124, 0.312)
**Sexism**									
Yes	0.257^*^ (−0.038, 0.551)	0.328^**^ (0.124, 0.533)	0.273^**^ (0.082, 0.464)	0.179^**^ (-−0.111, 0.468)	0.269^**^ (0.069, 0.47)	0.213^*^ (0.027, 0.4)	0.092 (−0.177, 0.362)	0.238^*^ **(0.042, 0.434)**	0.127 (−0.051, 0.306)
**Economic discrimination**									
Yes	0.190 (−0.134, 0.514)	−0.102 (−0.315, 0.112)	0.013 (−0.191, 0.217)	0.143 (−0.175, 0.461)	−0.093 (−0.301, 0.116)	0.031 (−0.168, 0.231)	0.097 (−0.202, 0.396)	−0.129 (−0.333, 0.075)	−0.03 (−0.222, 0.161)
**Cyberbullying**									
Yes	0.659^**^ (0.359, 0.959)	0.528^**^ (0.256, 0.801)	0.696^**^ (0.484, 0.909)	0.667^**^ (0.372, 0.962)	0.514^**^ (0.245, 0.782)	0.632^**^ (0.422,0.841)	0.415^**^ **(0.152, 0.768)**	0.288 (−0.023, 0.679)	0.521^**^ **(0.32, 0.722)**

* *P < 0.05*,

** *P < 0.01; The bold values indicate significant statistically differences. The reference category for the dependent variables were none (without family neglect or abuse, bullying at school, sex violence, sexism, economic discrimination, and Cyberbullying). Group 1: the adolescents in junior and senior school; Group 2: the adolescents in university/college. Model 1 adjusted for variables of the risk of social hurt, violence in schools, sex violence, sexism and economic discrimination and the Cyberbullying. Model 2 further adjusted for socio-demographic characteristics variables which including gender, nationality, regions, living conditions, and family support; Model 3 further adjusted for variables of lifestyle and health status*.

## Discussion

During the COVID-19 pandemic, isolation, contact restrictions, and economic shutdown impose a complete change to the psychosocial environment of affected countries (19). Higher prevalence of the SR (such as suicidal thoughts, suicide plans, SA, and self-harm) of adolescents in this study was found compared to a global adolescent suicidal rate for 2 years ago (20), and higher than the result of the finding of Chongqing in 2019 (21), especially of adolescents in junior and senior school. Although, it propounds the negative psychological effects of the epidemic on adolescents during the COVID-19 pandemic (22–24). Nevertheless, the reports suggest either no rise in the suicide rate or a fall in Japan (25), Norway (26), England (27), and Peru (28), in the early months of the pandemic. That may be the school closures have led to less academic and social pressure, and less peer conflict and bullying, which may have a substantial positive impact on the well-being of some vulnerable children and perhaps lead to lessened the suicidality rates (29). Although these findings are different, it also will be of great value and importance to explore how to decrease psychosocial risk factors associated with suicidality and to improve the mental health of a large groups of children.

Adolescents those suffered family neglect or abuse had a high score of SR, and the associations remained significant after adjusting for socio-demographic variables, whether those in college/university or those in middle school. During the COVID-19 pandemic period, adolescents were isolated in homes and spent more time with their families than before. Families will not only provide adolescents with financial and material resources, but also provide them with social support and social integration, which will have an important impact on their physical and mental health (30). The social distancing and school closures have made families play an even more important role in protecting teens' mental health during COVID-19. The previous studies showed that poor relationship between parents, family conflict, poor family cohesion, and strict parental discipline (such as corporal punishment) makes adolescents suffer from narcissistic trauma and leads to depression PTSD (post-traumatic stress disorder), and other mental illnesses, that significantly increase the risk of suicide among adolescents (31). These findings highlight the importance of family intervention as a possible strategy to prevent adolescents from committing suicide during isolation like during the COVID-19 pandemic period.

The association between SR and bullying at school, sex violence, and economic discrimination was not found among adolescents whether those in college/university or those in Middle School, after adjusting the confounding variables in this study. The school closures and social contacts are strongly limited, and out-of-home and leisure time activities are canceled during the COVID-19 pandemic (3), which may lead to less academic, social pressure, and less peer conflict and bullying and have a substantial positive impact on the well-being of some vulnerable children.

Life events before suicidal behavior are usually academic stressors (such as exam stress or bullying), family conflicts, disturbance, and other stressful life events (32). In this study, we found that the most obvious trauma that contributed to SR in junior and senior school adolescents was cyberbullying. To help school pupils continue their studies at home, the Ministry of Education (the MOE) opened an online learning platform offering a variety of courses based on commonly used textbooks in China (33). This may lead to a sudden ascent in internet-based life – combined with adolescents continually utilizing such stages could lead to a concerning spike in cyberbullying, which has a more serious impact on adolescents' suicidal behavior than other forms of violence (34, 35). According to a survey by the China Internet Network Information Center, the number of Internet users in China was 989 million in 2020, of which preadolescence and teenagers (10–19 years old) accounted for 13.5% of the total (36). Cyberbullying occurs when a child or teen uses the Internet, emails, text messages, instant messaging, social media websites, online forums, chat rooms, or other digital technology to harass, threaten, or humiliate another child or teen (37). Compared with junior high school students and senior high school students, college students have more rich social experience and a higher level of social education, which enables them of attaining a more rational mind and leads to their stronger immunity to cyberbullying. This study recommends that addressing younger adolescents, a better online environment can be created, and more acceptable online behavior education related to cyberbullying should be prioritized to reduce the risk of suicide.

Furthermore, a significant association between sexism and SR score was only found in the group of adolescents in college/university. This finding was similar to the previous study in Pakistan (38). Sexism refers to a belief that one sex is superior to the other and that the superior sex has endowments, rights, prerogatives, and status greater than those of the inferior sex, and results in discrimination in all areas of life and acts as a limiting factor in educational, professional, and psychological development (38). In recent years, the employment situation in the labor market in China has been severe, and the problem of gender differences has become more prominent (39). When adolescents attend college, they would increase the gender awareness and more complex interpersonal relationships (40), and face the society and labor market faster than high school students. Consequently, the concept of gender equality should be traded for everyone from an early age. Junior high schools, high schools, universities, and vocational higher education units can set up relevant compulsory courses.

The study was conducted within one province of China the samples may not represent all Chinese adolescents. As with all cross-sectional study designs, the causal nature of associations between different trauma and SR in this study cannot be determined. A longitudinal, multi-center sampling and multi-method design should be used in further study. Thus, the results should be interpreted with caution. Although anonymity, privacy, and confidentiality were assured, the self-report data may be impacted by social acceptability bias, particularly concerning sensitive questions such as a history of sexual assault. The COVID-19 is also a kind of trauma, and the community environment, sexual orientation, and other possible confounding variables may have impacted the SR, the interaction of COVID-19 and other different traumas should be considered in further studies.

## Conclusion

The findings highlight the different effects of sub-types of traumas on SR. The most potentially obvious trauma that contributed to SR in junior and senior school adolescents might be cyberbullying, and sexism contributed to the SR in those university students might be still serious during the COVID-19 pandemic period. Family neglect or abuse might be a detrimental factor in SR for adolescents whether those in junior school or those in university school. More attention should be paid to family education to decrease neglect or abuse and the SR during the COVID-19 prevention and control period. Intervention on cyberbullying and efforts should be prior conducted in adolescents in junior and senior school and the concept of gender equality should be traded for everyone from an early age to anti-sexism and suicide prevention.

## Data Availability Statement

The original contributions presented in the study are included in the article/supplementary material, further inquiries can be directed to the corresponding author/s.

## Ethics Statement

The studies involving human participants were reviewed and approved by Ethics Committee Review Committee of Chongqing Collaborative Innovation Center for Functional Food in Chongqing University of Education (202009HS01). Written informed consent to participate in this study was provided by the participants' legal guardian/next of kin.

## Author Contributions

YY and TW conducted statistical analyses of the data and prepared the draft manuscript. SW edited the manuscript. XZ and WL provide critical comments to the manuscript. All authors checked and proofread the final version of the manuscript.

## Funding

Chongqing Municipal Education Commission, Humanities and Social Sciences Research Project 2021 (21SKGH284).

## Conflict of Interest

The authors declare that the research was conducted in the absence of any commercial or financial relationships that could be construed as a potential conflict of interest.

## Publisher's Note

All claims expressed in this article are solely those of the authors and do not necessarily represent those of their affiliated organizations, or those of the publisher, the editors and the reviewers. Any product that may be evaluated in this article, or claim that may be made by its manufacturer, is not guaranteed or endorsed by the publisher.
